# tDCS Over DLPFC Leads to Less Utilitarian Response in Moral-Personal Judgment

**DOI:** 10.3389/fnins.2018.00193

**Published:** 2018-03-26

**Authors:** Haoli Zheng, Xinbo Lu, Daqiang Huang

**Affiliations:** ^1^Center for Economic Behavior and Decision-Making, Neuro & Behavior EconLab (NBEL), Zhejiang University of Finance and Economics, Hangzhou, China; ^2^Interdisciplinary Center for Social Sciences, Zhejiang University, Hangzhou, China; ^3^School of Economics, Zhejiang University of Finance and Economics, Hangzhou, China

**Keywords:** moral dilemma, dorsolateral prefrontal cortex, temporoparietal junction, transcranial direct current stimulation, dual-process theory, theory of mind

## Abstract

The profound nature of moral judgment has been discussed and debated for centuries. When facing the trade-off between pursuing moral rights and seeking better consequences, most people make different moral choices between two kinds of dilemmas. Such differences were explained by the dual-process theory involving an automatic emotional response and a controlled application of utilitarian decision-rules. In neurocognitive studies, the bilateral dorsolateral prefrontal cortex (DLPFC) has been demonstrated to play an important role in cognitive “rational” control processes in moral dilemmas. However, the profile of results across studies is not entirely consistent. Although one transcranial magnetic stimulation (TMS) study revealed that disrupting the right DLPFC led to less utilitarian responses, other TMS studies indicated that inhibition of the right DLPFC led to more utilitarian choices. Moreover, the right temporoparietal junction (TPJ) is essential for its function of integrating belief and intention in moral judgment, which is related to the emotional process according to the dual-process theory. Relatively few studies have reported the causal relationship between TPJ and participants' moral responses, especially in moral dilemmas. In the present study, we aimed to demonstrate a direct link between the neural and behavioral results by application of transcranial direct current stimulation (tDCS) in the bilateral DLPFC or TPJ of our participants. We observed that activating the right DLPFC as well as inhibiting the left DLPFC led to less utilitarian judgments, especially in moral-personal conditions, indicating that the right DLPFC plays an essential role, not only through its function of moral reasoning but also through its information integrating process in moral judgments. It was also revealed that altering the excitability of the bilateral TPJ using tDCS negligibly altered the moral response in non-moral, moral-impersonal and moral-personal dilemmas, indicating that bilateral TPJ may have little influence over moral judgments in moral dilemmas.

## Introduction

The nature of moral judgment has been debated for centuries. To analyze the moral brain of humans, a valid measurement is by observing participants' responses to moral dilemmas, which present a story involving a trade-off between pursuing moral rights and seeking better consequences (Borg et al., 2006). When people make moral judgments in conflicts between harm and moral rights, both reason and emotion are considered important forces driving moral judgments. Greene et al. (2001) classified moral dilemmas into two categories: moral-impersonal dilemmas (e.g., switch dilemma) and moral-personal dilemmas (e.g., footbridge dilemma). Most people may find it appropriate to save five lives at the expense of one by turning a switch in a classic switch dilemma (Thomson, 1986), whereas in a footbridge dilemma, they may consider it inappropriate to push a stranger off the footbridge in order to stop the train, which may also save the lives of five people (Greene et al., 2001). By considering both reason and emotion as essential forces in moral decisions, such differences in moral responses are explained by the dual-process theory (Greene et al., 2001, 2004). According to the dual-process theory, moral decisions are made involving an automatic emotional response and a controlled application of rational utilitarian decision-rules. The moral emotional response is considered too strong to be overwhelmed by the cognitive reasoning process in moral-personal dilemmas while in contrast, participants may favor the utilitarian choice in moral-impersonal dilemmas because the weaker emotional response is manipulated by rational cognitive control (Greene, 2007).

The cognitive reasoning process has been directly related to the involvement of the dorsolateral prefrontal cortex (DLPFC) in moral decisions (Greene et al., 2001, 2004). According to the dual-process theory, the right DLPFC may lead to utilitarian choices through its influence over the cognitive rational control process. However, the profile of results across studies is not entirely consistent. It has been revealed that damage to the frontal cortex leads to utilitarian moral judgments that rely solely on best results. Recent transcranial magnetic stimulation (TMS) studies have also raised questions regarding the role of the right DLPFC restricted to rational cognitive control. Using low-frequency repetitive transcranial magnetic stimulation (rTMS), Knoch et al. (2006) revealed that disrupting the function of participants' right DLPFC reduced the rejection rates of their partners' intentionally unfair offers, leading to a more utilitarian judgment in an economic interaction. Moreover, Tassy et al. (2011) applied rTMS over participants' right DLPFC while subjecting them to moral tasks and demonstrated that disrupting the right DLPFC alters moral judgment, increasing the probability of utilitarian responses. These TMS studies indicated that suppressing the right DLPFC may result in more utilitarian judgments, suggesting that the right DLPFC function not only participates in a rational cognitive control process but also integrates emotions in moral judgments, especially in high-conflict moral dilemmas (Tassy et al., 2011). In contrast, Jeurissen et al. (2014) revealed that TMS-induced disruption of the DLPFC in moral-personal decisions leads to less utilitarian decisions, which supported the dual-process theory. The contradiction of the observations in these two studies may due to their relatively small sample sizes which may affect the robustness of the results.

Emotional response is associated with the bilateral temporoparietal junction (TPJ), which plays a significant role in the process of belief attribution in moral judgments (Ruby and Decety, 2001; Vogeley et al., 2001; Gallagher and Frith, 2003; Schleim et al., 2010; Mai et al., 2016). When individuals make moral decisions, the bilateral TPJ is centrally involved in understanding others by reasoning about the content of mental states (Saxe and Kanwisher, 2003; Jeurissen et al., 2014). Previous studies have demonstrated people making moral judgments depending more substantially on beliefs and intentions rather than on results and consequences (Surber, 1977; Shultz et al., 1986; Baird and Moses, 2001; Baird and Astington, 2004). This type of behavior may be interpreted by the theory of mind: the ability to attribute mental states, such as beliefs and intentions, to moral agents, which also play a crucial role in the process of moral judgment (Borg et al., 2006; Cushman et al., 2006; Young et al., 2007). The right TPJ is associated with beliefs because its activity was observed to be significantly higher when participants read false belief stories (Sommer et al., 2007; Aichhorn et al., 2009; Young and Dodell, 2010). Using TMS, Young et al. (2010) demonstrated a direct causal link between the disruption of the right TPJ and the decreasing influence of beliefs in moral judgment. More recently, Sellaro et al. (2015) demonstrated that anodal stimulation of the right TPJ enhanced the role of belief in moral judgment, suggesting that the right TPJ integrates beliefs and intentions into participants' moral judgments. Using transcranial direct current stimulation (tDCS), Ye et al. (2015) revealed that the bilateral TPJ is indispensable for integrating intentions in moral judgment. Leloup et al. (2016) also indicated that the right TPJ may play multiple roles in moral cognition, in relation to the methodological aspects of the use of tDCS. However, the moral tasks in these studies were moral judgments involving both intentions and consequences rather than moral dilemmas. Using moral dilemma tasks, Jeurissen et al. (2014) revealed that disrupting the function of TPJ affects only moral-impersonal conditions in moral dilemmas.

Although cognitive reasoning and emotional processes, as identified in the dual-process theory, have been associated with the activity of the DLPFC and TPJ (Greene et al., 2001, 2004), no conclusive results have been demonstrated in previous neural imaging and stimulation studies. In the current study, using tDCS which allows cortical excitability to be directly manipulated, we aimed to investigate whether modulating the excitability of the bilateral DLPFC (or TPJ) can directly influence our participants' moral judgments by affecting their cognitive reasoning or emotional processes. Furthermore, we enlarged the sample size to 20 participants in each group with total of 100 valid subjects to examined the robustness of the double-dissociation effect between DLPFC and TPJ on the outcome of a moral decision. The casual relationship between the activity of bilateral DLPFC (or TPJ) and individuals' moral judgments may be revealed by comparing their judgments among different types of stimulations of the bilateral DLPFC (or TPJ).

## Materials and methods

### Subjects

One hundred right-handed healthy subjects (mean age 21.4 years, ranging from 17 to 30 years; 52 females) with no history of neurological or psychiatric problems participated in the study for payment. All the participants were naïve to tDCS and moral judgment tasks, had normal or corrected-to-normal vision, and provided their written informed consent, which was approved by the Zhejiang University ethics committee. The entire experiment lasted approximately 30 min, and each participant received a payment of 50 RMB Yuan (approximately 7.576 US dollars) upon completion of their tasks. None of the participants reported any adverse side effects concerning pain on the scalp or headaches after the experiment.

### tDCS

For tDCS, a weak direct current was applied to the scalp via two saline-soaked surface sponge electrodes (35 cm^2^). The current was constant and was delivered by a battery-driven stimulator (Multichannel noninvasive wireless tDCS neurostimulator, Starlab, Barcelona, Spain). It was adjusted to induce cortical excitability of the target area without any physiological damage to the participants. Various configurations of the current had various effects on cortical excitability; anodal stimulation enhanced cortical excitability, whereas cathodal stimulation suppressed it (Nitsche and Paulus, 2000).

**Figure 1:**
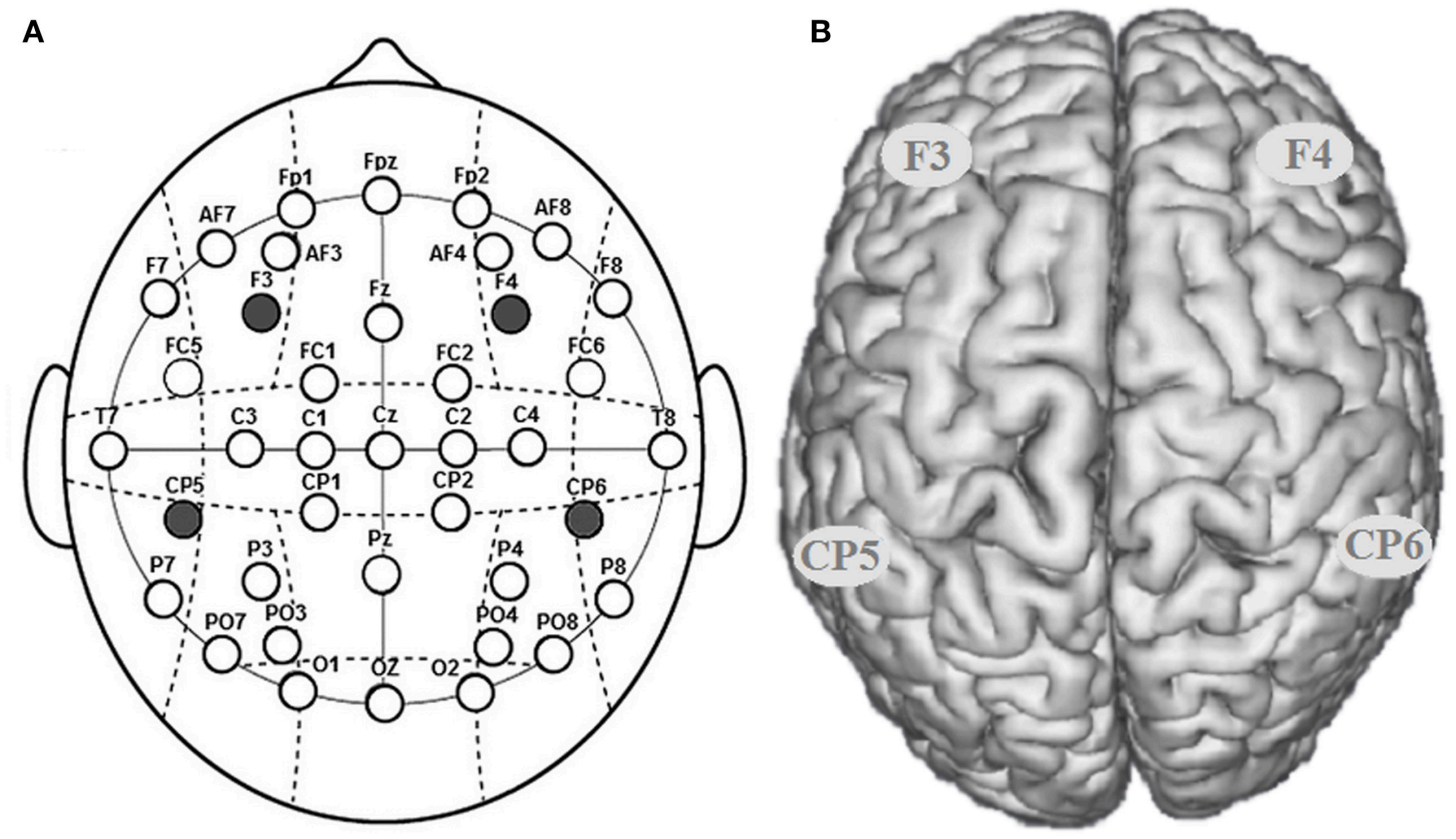
Locations of the electrode positions. **(A)** Schematic of the electrode positions DLPFC (F3, F4) and TPJ (CP5, CP6) based on the international EEG 10-20 system. **(B)** Locations of the DLPFC (F3, F4) and the TPJ (CP5, CP6) of the human brain.

**Figure 2:**
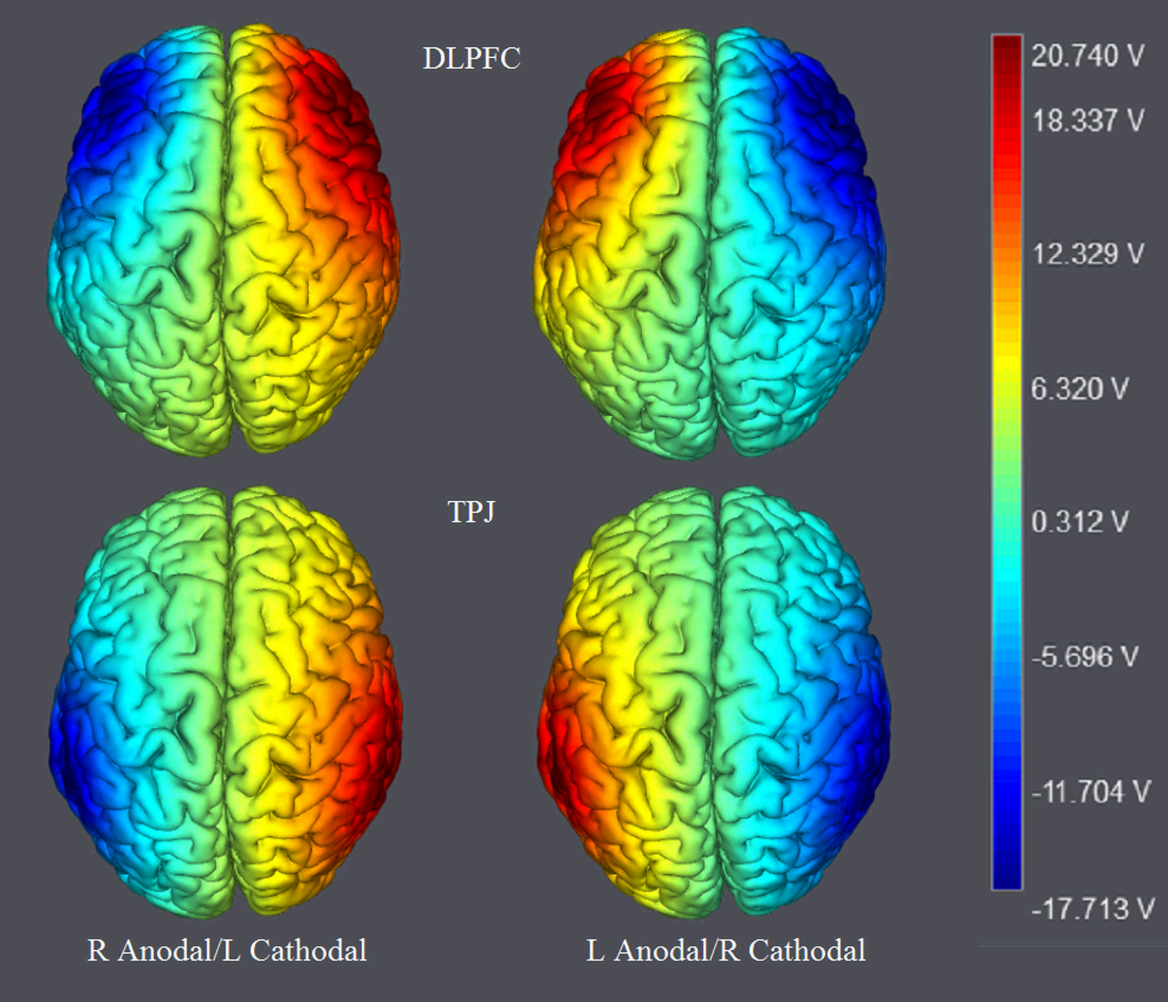
The stimulation modes of tDCS treatments. The axis represents the range of input voltage from −17.713 to 20.740 V.

The participants were randomly assigned to receive right anodal/left cathodal tDCS over DLPFC (*n* = 20, 12 females), left anodal/right cathodal tDCS over DLPFC (*n* = 20, 10 females), right anodal/left cathodal tDCS over TPJ (*n* = 20, 11 females), left anodal/right cathodal tDCS over TPJ (*n* = 20, 9 females) or sham stimulation (*n* = 20, 10 females). For right anodal/left cathodal stimulation over DLPFC, the anodal electrode was placed over the right DLPFC at the F4 position according to the international EEG 10/20 system, whereas the cathodal electrode was placed over the left DLPFC at the F3 position. For left anodal/right cathodal stimulation, the placement was reversed. For right anodal/left cathodal and left anodal/right cathodal tDCS stimulations over TPJ, the placement of electrodes was identical to those over DLPFC (Figures 1, 2). For sham stimulation, the procedures were the same (the placement of electrodes was either over the bilateral DLPFC or over the bilateral TPJ), but the current lasted for only the first 30 s. The participants may have felt the initial itching, but there was actually no current for the rest of the stimulation. This method of sham stimulation has been shown to be reliable (Gandiga et al., 2006). The current was constant and of 2 mA in intensity, with a 30 s ramp up and down; the safety and efficiency of this stimulation has been demonstrated in previous studies. Before the moral judgment task, the laboratory assistant put a tDCS device on the participant's head for stimulation. After 20 min of stimulation, the participant was then asked to complete a moral judgment task.

### Task and procedure

After the participants received tDCS stimulation for 20 min (single-blinded, sham-controlled), they completed a moral judgment task (the computer program for the task was written in visual C#), which was similar to Greene's design (Greene et al., 2001). The moral dilemma task involved 12 stories, including 4 non-moral dilemmas, 4 moral-impersonal dilemmas and 4 moral-personal dilemmas (Supplementary Material). Moral dilemmas were presented in a pseudorandom order, the order of stories counterbalanced across runs, ensuring that same type of moral dilemma was never immediately repeated. Each participant read the 12 stories as text, then rated the degree of appropriateness of the protagonists' actions on a 10-point scale (1 = completely appropriate; 10 = completely inappropriate). Upon completing the moral task, participants had to complete a questionnaire before receiving their payments.

## Results

The reaction time and the response rating data were statistically evaluated using the SPSS software (version 22, SPSS Inc., Chicago, IL, USA). The significance level was set at 0.05 for all analyses.

### Response

Response ratings from the right anodal/left cathodal tDCS over TPJ, left anodal/right cathodal tDCS over TPJ, right anodal/left cathodal tDCS over DLPFC, left anodal/right cathodal tDCS over DLPFC and sham groups were analyzed by repeated measures analyses of variance (ANOVAs) with dilemma type as a within-subject factor and tDCS stimulation type as a between-subject factor. A significant influence of dilemma type was observed [*F*_(2, 790)_ = 673.587, *p* < 0.001, partial η^2^ = 0.630]. The utilitarian responses of protagonists were considered more inappropriate in moral-personal dilemmas (average rating of 7.54) than those in moral-impersonal dilemmas (average rating of 4.08, *p* < 0.001) or in non-moral dilemmas (average rating of 1.81, *p* < 0.001).

**Figure 3:**
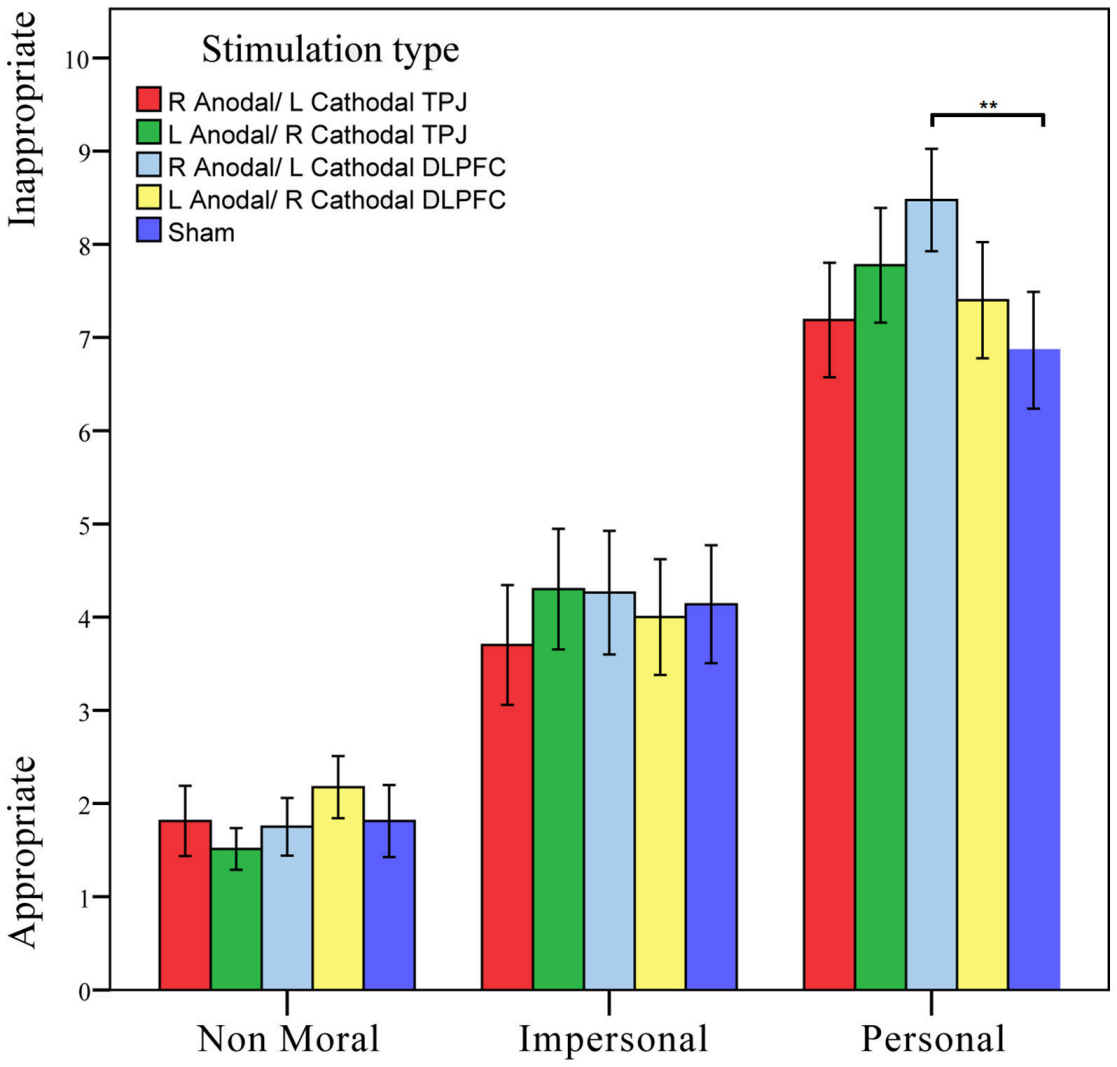
Data of moral response ratings. The moral response ratings of participants with moral-personal, moral-impersonal and non-moral conditions across stimulations. Error bars indicate 95% confidence intervals. Asterisks indicate statistical significance of difference between treatments.

Notably, there was a significant interaction effect involving the dilemma type and stimulation type [*F*_(8, 790)_ = 2.633, *p* = 0.008, partial η^2^ = 0.026]. *Post hoc* analyses (Bonferroni) revealed that in the personal dilemma tasks, the response ratings obtained in the right anodal/left cathodal DLPFC group (average rating of 8.475) were significantly higher than those obtained in the sham group (average rating of 6.863, *p* = 0.002). No other significant effects were observed in the impersonal dilemma or non-moral dilemma tasks (Figure 3).

### Reaction times

All trials in which reaction times were too long (>30 s) were excluded from data analysis (Jeurissen et al., 2014). Reaction times obtained following right anodal/left cathodal tDCS over TPJ, left anodal/right cathodal tDCS over TPJ, right anodal/left cathodal tDCS over DLPFC, left anodal/right cathodal tDCS over DLPFC and from the sham groups were analyzed by repeated measures analyses of variance (ANOVAs) with the dilemma type as a within-subject factor and tDCS stimulation type as a between-subject factor. No significant influence of tDCS stimulation type was observed [*F*_(4, 369)_ = 0.710, *p* = 0.585, partial η^2^ = 0.008]. No significant interaction effect involving dilemma type and stimulation type was observed [*F*_(8, 738)_ = 0.585, *p* = 0.790, partial η^2^ = 0.006]. The reaction times in moral-impersonal dilemmas (mean = 8,357ms) were significantly higher than that in moral-personal dilemmas (mean = 7,098, *p* < 0.007) or in non-moral dilemmas (mean = 7,072, *p* < 0.006). Crucially, there was a significant negative correlation between reaction times and response ratings within the moral-personal condition (coefficient = −0.218, *p* < 0.001, Pearson correlation). There was also a significant positive correlation between reaction times and response ratings within the non-moral condition (coefficient = 0.189, *p* < 0.001, Pearson correlation). No significant correlation between reaction times and response ratings within the moral-impersonal condition was observed (coefficient = −0.218, *p* = 0.424, Pearson correlation) (Table 1).

**Table 1:** The mean and SD of reaction time across moral contents and stimulations.

**Moral Content**	**Stimulation type**	**R Anodal/L Cathodal over DLPFC**	**L Anodal/R Cathodal over DLPFC**	**R Anodal/L Cathodal over TPJ**	**L Anodal/R Cathodal over TPJ**	**Sham**
Non-moral	Mean (ms)	6994.535	7092.519	6309.587	7433.849	7528.311
	SD	577.772	589.065	609.454	589.065	605.206
Moral impersonal	Mean (ms)	7619.179	8485.320	8869.920	8966.988	7843.793
	SD	699.502	713.174	737.859	713.174	732.717
Moral personal	Mean (ms)	6700.983	7595.328	6997.196	7532.256	6663.313
	SD	607.357	619.229	640.662	619.229	636.197

## Discussion

### DLPFC and moral dilemma

The dual-process theory hypothesizes that in high-conflict moral-personal dilemmas, stronger rational cognitive control is required to overrule the initial emotional impulse. Using TMS, Jeurissen et al. (2014) supported the dual-process theory by revealing that disruption of the right DLPFC leads to less utilitarian choices. It was explained by the dual-process theory that the DLPFC is majorly involved in “rational cognitive” control superseding emotional impulse, which is not strong enough. In contrast, Tassy et al. (2011) observed that disrupting the function of the right DLPFC leads to more utilitarian choices in moral dilemmas. Moreover, it has also been claimed that the dual-process theory may not be sufficient to explain various aspects of moral cognitions (Buckholtz and Marois, 2012; Van Bavel et al., 2015) suggested that the role of DLPFC in prosocial behaviors may be not solely restricted to its rational cognitive control process. In the current study, we observed that activation of the right DLPFC and inhibition of the left DLPFC by tDCS led to less utilitarian choices, especially in moral-personal conditions, supporting the claim that apart from its function in rational cognitive control process, the right DLPFC also plays an essential role in integrating emotional information in moral judgments. Such an emotional integration process was only observed in high-conflict dilemmas, such as moral-personal dilemmas. When confronting moral-personal dilemmas, the conflict of pursuing moral rights and seeking better consequences was stronger than moral-impersonal and non-moral dilemmas. In moral-personal dilemmas, the strengthened excitability of the right DLPFC weighed more on the initial emotional impulse through its emotion integrating process, resulting in less utilitarian moral response. In contrast, in moral-impersonal dilemmas, when the conflict of moral rights and better results was much weaker than in moral-personal condition, the enhancement of right DLPFC negligibly altered moral decisions.

Observations of the current study may be explained by the hypothesis provided by Buckholtz and Marois (2012). Buckholtz and Marois (2012) revealed that the dual-process theory could not completely explain the role of DLPFC in altruistic punishment games. According to the dual-process theory, if the role of the right DLPFC is solely in rational cognitive control process, its inhibition may result in less utilitarian choices in punishment games, which is not consistent with the finding that this brain region is activated to a greater extent when participants decide to punish protagonists in third-party interactions (Buckholtz et al., 2008). The role of the right DLPFC may be that it selects a specific response from among possible response options by integrating information about harm and blame with context-specific rules. In the current case of moral decisions, the more appropriate explanation regarding the role of the right DLPFC may be that it selects a specific moral response from these possible options by integrating information about moral rights and utilitarian consequences with dilemma-specific contents following moral rules.

fMRI studies revealed that the excitability of the frontal cortex was higher in moral-personal situations than that in non-moral and moral-impersonal situations (Greene et al., 2001, 2004). More recently, Jeurissen et al. (2014) revealed that TMS-induced disruption of the DLPFC only affects moral-personal decisions, leading to less utilitarian moral choices. However, several TMS studies, using moral decision tasks or prosocial economic games measuring fairness of participants (e.g., Ultimatum game), indicated that disruption of the DLPFC may result in more utilitarian choices (Knoch et al., 2006; Tassy et al., 2011). Moreover, whether enhancing the activity of bilateral DLPFC alters participants' non-moral, moral-impersonal and moral-personal decisions remains unknown. In the current study, we observed that modulating the excitability of the bilateral DLPFC altered the participants' moral judgments, especially in moral-personal situations. Our results confirmed the casual relationship between the activity of DLPFC and the moral decisions of participants in moral-personal conflicts. No such causal relationship between the activity of DLPFC and the moral choices in moral-impersonal or non-moral conflicts was observed.

Moreover, the left DLPFC has also been revealed that enhancing the activity of this brain region may induce a shift in moral judgment toward more non-utilitarian actions (Kuehne et al., 2015). No such effect was observed in our study. The difference between the findings of our study and the observations of the previous study may due to the variety in experimental designs and the stimulation locations. Kuehne et al. (2015) placed the active electrode over the left DLPFC with the reference electrode over the right parietal cortex, while in the current study the target and reference electrodes were placed over the bilateral DLPFC. Kuehne et al. (2015) performed a within-subject study and we performed a between-subject study which may also lead to inconsistent findings. In addition, our finding may also be the result of a combination stimulation effect over the bilateral DLPFC. The function of the left DLPFC may be justified through unilateral stimulation in further studies.

### TPJ and moral dilemma

According to the dual-process theory, the emotional response may have been influenced by the TPJ through its function described in the theory of mind. TMS and tDCS studies have demonstrated that altering the activity of TPJ may change moral decisions, especially in conditions involving beliefs and intentions (Young et al., 2007; Young and Saxe, 2008; Sellaro et al., 2015; Ye et al., 2015). Jeurissen et al. (2014) revealed that disruption of TPJ leads to less utilitarian choices, especially in moral-impersonal dilemmas. In the current study, we observed that neither enhancing nor reducing the excitability of bilateral TPJ altered moral decisions, regardless of the dilemmas being moral-personal, moral-impersonal or non-moral conditions. Since the responses of participants confronting moral dilemmas were not identical to the moral judgments involving beliefs and intentions, the mechanism of altering moral decisions through the theory of mind in other moral judgments may not be available for the moral response in moral dilemmas. The observations in our current study do not support the findings of previous studies. However, the findings in the current study may be due to the combination of bilateral anodal and cathodal tDCS stimulations of TPJ, and further study is needed focusing on separating the influences of the left and right TPJ to discuss the functions of these brain regions, respectively.

To sum up, we conclude that in moral dilemmas, altering the activation of the bilateral DLPFC may change moral responses by altering its information integrating process in moral decisions, especially in high conflict moral-personal dilemmas, while modulating the excitability of TPJ has no significant effect over moral responses through its function, as described in the theory of mind.

### Reaction times and moral responses

The observation that those saying more “appropriate” to moral-personal dilemmas exhibit longer reaction times also indicates that they experienced greater emotional interference in high-conflict moral dilemmas (Greene et al., 2001). On contrast, no such correlation between reaction times and response ratings within the moral-impersonal condition was observed and the data within the non-moral condition exhibit a significant opposite direction. The relationship between behavioral moral responses and respective reaction times further proved that the emotional interference may play an essential role in moral dilemmas, especially in moral-personal dilemmas.

### Limitations

One limitation of the current study is that although our findings in the DLPFC confirmed that modulating the excitability of the right DLPFC altered participants' moral judgments through its function of moral information integrating process, the mechanism underlying the bilateral DLPFC altering the moral response in moral-personal dilemmas remains to be revealed and discussed. Another deficiency of our study is that the results of our experiment were based on stimulation of the bilateral DLPFC or TPJ, and may reflect the combination of bilateral anodal and cathodal tDCS stimulations. Future studies focused on separating the influences of the left and right DLPFC or TPJ and discussing the functions of these brain regions, are required.

## Conclusion

In summary, our findings provide important information regarding the impact of tDCS on the DLPFC of healthy participants, especially with respect to moral-personal dilemmas. Activating the right DLPFC while inhibiting the left DLPFC by tDCS may lead to less utilitarian responses in moral judgment, especially in moral-personal dilemmas, supporting the claim that the right DLPFC plays an essential role, not only through its function of moral reasoning but also through its emotional information integrating process in moral judgments. Moreover, neither enhancing nor reducing the excitability of the bilateral TPJ altered participants' moral decisions, regardless of the dilemmas being moral-personal, moral-impersonal or non-moral conditions, indicating that the bilateral TPJ may have little influence over moral judgments in moral dilemmas.

## Ethics statement

This study was carried out in accordance with the recommendations of the guideline of tDCS experiment, Zhejiang University ethics committee with written informed consent from all subjects. All subjects gave written informed consent in accordance with the Declaration of Helsinki. The protocol was approved by the Zhejiang University ethics committee.

## Author contributions

HZ, XL, and DH designed experiment; HZ and DH performed experiment; HZ, XL, and DH analyzed data; HZ drew figures; HZ, XL, and DH wrote the manuscript; HZ, XL, and DH revised the manuscript and HZ, XL, and DH finally approved the version to be published.

### Conflict of interest statement

The authors declare that the research was conducted in the absence of any commercial or financial relationships that could be construed as a potential conflict of interest.
