# Supplementary material for: tDCS Over DLPFC Leads to Less Utilitarian Response in Moral-Personal Judgment
Source: Front Neurosci. 2018 Mar 26;12:193. doi: 10.3389/fnins.2018.00193 (PMC5879123; doi:10.3389/fnins.2018.00193)
Supplement: Supplementary file 1 [file DataSheet1.DOCX]

The dilemmas employed in Experiment are presented below. The types and names of the dilemmas were given for the convenience of the experimenters and were not seen by subjects. Moral dilemmas were presented in a pseudorandom order, the order of conditions counterbalanced across runs, ensuring that same type of moral dilemma was never immediately repeated.

Non-Moral Dilemmas:

1. Brownies

You have decided to make a batch of brownies for yourself. You open your recipe book and find a recipe for brownies.

The recipe calls for a cup of chopped walnuts. You don't like walnuts, but you do like macadamia nuts. As it happens, you have both kinds of nuts available to you.

Is it appropriate for you to substitute macadamia nuts for walnuts in order to avoid eating walnuts?

2. Train or Bus

You need to travel from New York to Boston in order to attend a meeting that starts at 2:00 PM. You can take either the train or the bus.

The train will get you there just in time for your meeting no matter what. The bus is scheduled to arrive an hour before your meeting, but the bus is occasionally several hours late because of traffic. It would be nice to have an extra hour before the meeting, but you cannot afford to be late.

Is it appropriate for you to take the train instead of the bus in order to ensure you not being late for your meeting?

3. Computer

You are looking to buy a new computer. At the moment the computer that you want costs $1000. A friend who knows the computer industry has told you that this computer's price will drop to $500 next month.

If you wait until next month to buy your new computer you will have to use your old computer for a few weeks longer than you would like to. Nevertheless you will be able to do everything you need to do using your old computer during that time.

Is it appropriate for you to use your old computer for a few more weeks in order to save $500 on the purchase of a new computer?

4. Standard Turnips

You are a farm worker driving a turnip-harvesting machine. You are approaching two diverging paths.

By choosing the path on the left you will harvest ten bushels of turnips. By choosing the path on the right you will harvest twenty bushels of turnips. If you do nothing your turnip-harvesting machine will turn to the left

Is it appropriate for you to turn your turnip-picking machine to the right in order to harvest twenty bushels of turnips instead of ten?

Moral Impersonal Dilemmas:

1. Standard Trolley

You are at the wheel of a runaway trolley quickly approaching a fork in the tracks. On the tracks extending to the left is a group of five railway workmen. On the tracks extending to the right is a single railway workman.

If you do nothing the trolley will proceed to the left, causing the deaths of the five workmen. The only way to avoid the deaths of these workmen is to hit a switch on your dashboard that will cause the trolley to proceed to the right, causing the death of the single workman.

Is it appropriate for you to hit the switch in order to avoid the deaths of the five workmen?

2. Donation

You are at home one day when the mail arrives. You receive a letter from a reputable international aid organization. The letter asks you to make a donation of two hundred dollars to their organization.

The letter explains that a two hundred-dollar donation will allow this organization to provide needed medical attention to some poor people in another part of the world.

Is it appropriate for you to not make a donation to this organization in order to save money?

3. Standard Fumes

You are the late-night watchman in a hospital. Due to an accident in the building next door, there are deadly fumes rising up through the hospital's ventilation system. In a certain room of the hospital are three patients. In another room there is a single patient. If you do nothing the fumes will rise up into the room containing the three patients and cause their deaths.

The only way to avoid the deaths of these patients is to hit a certain switch, which will cause the fumes to bypass the room containing the three patients. As a result of doing this the fumes will enter the room containing the single patient, causing his death.

Is it appropriate for you to hit the switch in order to avoid the deaths of the three patients?

4. Environmental Policy

You are a member of a government legislature. The legislature is deciding between two different policies concerning environmental hazards.

Policy A has a 90% chance of causing no deaths at all and has a 10% chance of causing 1000 deaths. Policy B has a 92% chance of causing no deaths and an 8% chance of causing 10,000 deaths.

Is it appropriate for you to vote for Policy B over Policy A?

Moral Personal Dilemmas:

1. Transplant

You are a doctor. You have five patients, each of whom is about to die due to a failing organ of some kind. You have another patient who is healthy.

The only way that you can save the lives of the first five patients is to transplant five of this young man's organs (against his will) into the bodies of the other five patients. If you do this, the young man will die, but the other five patients will live.

Is it appropriate for you to perform this transplant in order to save five of your patients?

2. Lifeboat

You are on a cruise ship when there is a fire on board, and the ship has to be abandoned. There are 6 passengers and the lifeboats are carrying many more people than they were designed to carry. The lifeboat you're in is sitting dangerously low in the water-a few inches lower and it will sink.

The seas start to get rough, and the boat begins to fill with water. It seems to you that there is only one way to stop the boat from sinking, and that is to start throwing one passenger overboard.

Is it appropriate for you to throw one of your fellow passengers overboard in order to save the remaining five passengers?

3. Footbridge

A runaway trolley is heading down the tracks toward five workmen who will be killed if the trolley proceeds on its present course. You are on a footbridge over the tracks, in between the approaching trolley and the five workmen. Next to you on this footbridge is a stranger who happens to be very large.

The only way to save the lives of the five workmen is to push this stranger off the bridge and onto the tracks below where his large body will stop the trolley. The stranger will die if you do this, but the five workmen will be saved.

Is it appropriate for you to push the stranger on to the tracks in order to save the five workmen?

4. Architect

You are a young architect visiting one of your construction sites with your boss. Your boss is a despicable individual who makes everyone around him miserable including you.

It occurs to you that if you were to push him off of the building you are inspecting he would fall to his death and everyone would think it was an accident.

Is it appropriate for you to push your boss off of the building in order to get him out of your life?
